# Low Thalamic NAA-Concentration Corresponds to Strong Neural Activation in Working Memory in Kleine-Levin Syndrome

**DOI:** 10.1371/journal.pone.0056279

**Published:** 2013-02-25

**Authors:** Patrick Vigren, Anders Tisell, Maria Engström, Thomas Karlsson, Olof Leinhard Dahlqvist, Peter Lundberg, Anne-Marie Landtblom

**Affiliations:** 1 Department of Clinical and Experimental Medicine (IKE)/Neuroscience, Linköping University, and Department of Neurosurgery, County Council of Östergötland, Linköping, Sweden; 2 Center of Medical Image Science and Visualization (CMIV), Linköping University, Linköping, Sweden; 3 Radiation Physics, Department of Medical and Health Sciences, Linköping University, Linköping, Sweden; 4 Radiology, Department of Medical and Health Sciences, Linköping University, Linköping, Sweden; 5 Department of Behavioural Science and Learning, Linköping University, Linköping, Sweden; 6 Radiation Physics, Department of Medical and Health Sciences, Linköping University, and Department of Radiation Physics UHL, County Council of Östergötland, Linköping, Sweden; 7 Radiology, Department of Medical and Health Sciences, Linköping University, and Department of Radiology UHL, County Council of Östergötland, Linköping, Sweden; 8 Department of Clinical and Experimental Medicine (IKE)/Neuroscience, Linköping University, and Department of Neurology, County Council of Östergötland, Linköping, Sweden; University of Manchester, United Kingdom

## Abstract

**Background:**

Kleine Levin Syndrome (KLS) is a rare disorder of periodic hypersomnia and behavioural disturbances in young individuals. It has previously been shown to be associated with disturbances of working memory (WM), which, in turn, was associated with higher activation of the thalamus with increasing WM load, demonstrated with functional magnetic resonance imaging (fMRI). In this study we aimed to further elucidate how these findings are related to the metabolism of the thalamus.

**Methods:**

fMRI and magnetic resonance spectroscopy were applied while performing a WM task. Standard metabolites were examined: n-acetylaspartate (NAA), myo-inositol, choline, creatine and glutamate-glutamine. Fourteen KLS-patients and 15 healthy controls participated in the study. The patients with active disease were examined in asymptomatic periods.

**Results:**

There was a statistically significant negative correlation between thalamic fMRI-activation and thalamic NAA, i.e., high fMRI-activation corresponded to low NAA-levels. This correlation was not seen in healthy controls. Thalamic levels of NAA in patients and controls showed no significant differences between the groups. None of the other metabolites showed any co-variation with fMRI-activiation.

**Conclusion:**

This study shows negative correlation between NAA-levels and fMRI-activity in the left thalamus of KLS-patients while performing a WM task. This correlation could not be found in healthy control subjects, primarily interpreted as an effect of increased effort in the patient group upon performing the task. It might indicate a disturbance in the neuronal networks responsible for WM in KLS patients, resulting in higher effort at lower WM load, compared with healthy subjects. The general relationship between NAA and BOLD-signal is also discussed in the article.

## Introduction

The Kleine Levin Syndrome (KLS) was first described systematically during the early 20^th^ century [1], [2]. Presenting mainly during adolesence, it is a rare disorder of periodic hypersomnia associated with behavioral disturbances such as hyperphagia, irritability and hypersexuality. It has a mean duration of eight years and the mean duration of episodes is typically ten days recurring every 3.5 months, with vast inter- and intraindividual variation [3]. At this point, no apparent etiology of the disorder has been detected, and no convincing treatment has been found. Traditionally, patients are considered to have normal function between hypersomnia periods, regarding sleep patterns [4], [5] as well as cognitive function. In contrast to this view, we have shown disturbances of complex working memory (WM) that sometimes is long lasting, perhaps even permanent [3], [6]–[8]. Thus, KLS is an *in vivo* model of the hypothetical association of (periodic) sleep regulation and WM deficit, an association not yet thoroughly examined.

In our KLS patient group, including patients from Nordic countries, single photon emission computed tomography (SPECT) has shown that the patients often have hypoperfusion in the fronto-temporal areas [6], including areas traditionally linked to WM function [7]. We have also reported that the left lateral prefrontal cortex showed larger Blood Oxygen Level Dependent (BOLD) activation and that the cingulate cortex showed less BOLD-activation in KLS-patients while performing a verbal WM task during functional magnetic resonance imaging (fMRI), compared to healthy control subjects. However, the patients showed increased activity in the left thalamus [9]. In other reports, thalamic hypoperfusion has been observed during episodes, not persisting between hypersomnia epidodes, in KLS-patients using SPECT [10]. Other conditions with well-described WM impairment, such as schizophrenia, have shown a similar thalamic activation pattern during WM tasks [11].

Magnetic Resonance Spectroscopy (MRS) is a non-invasive method for investigating brain metabolites *in vivo*. Some of the most significant metabolites determined using this method are total N-acetyl compounds (tNA) consisting of N-acetylaspartate (NAA) and N-acetylaspartateglutamate (NAAG). These compounds are regarded as markers of neuronal health, viability and number. Total creatine (tCr) consists of creatine and phosphocreatine and reflects energy deposits and metabolism in general. Lipids reflect neuron membrane breakdown. Choline is considered a marker of cellular turnover in malignancy and inflammation. Lactate is a marker for anaerobic metabolism by several underlying pathologies. Myo-inositol (mIns) is a glial marker. Glutamat/glutamine (Glx) is an excitatory neurotransmittor, elevated in several metabolic cerebral conditions [12]–[14].

Concentrations of the different brain metabolites reflect the pathological status of the tissue and they are modulated in a range of neurological disorders. In idiopathic normal pressure hydrocephalus – a condition associated with WM impairment - there is a marked reduction of NAA in the thalamus [15]. In schizophrenia, also associated with impaired WM, lower NAA concentration compared with healthy controls has been observed [16], although another study has showed no correlation between verbal memory performance and thalamic NAA_levels [17]. Regarding MRS investigations in KLS-patients there is, to the best of our knowledge, only a single case report. In this patient, with classical KLS, the authors performed MRS investigations of both hypothalami and thalami inter- and intraictally, *i.e.* between and during hypersomnic periods. The authors found a difference between the two examinations in the ratios of tNA/Cr and Glx/Cr. They interpreted their findings as indicative of a thalamic dysfunction and that the variation in Glx/Cr-ratio was due to depolarisation block and post-excitatory suppression of various thalamic nuclei [18]. A variability in thalamic glutamine between asymptomatic and symptomatic periods has also been shown in a recent case report of a patient also showing varying fMRI patterns to tone stimuli and reduced thalamic perfusion during a symptomatic period [19].

As the thalamus also is involved in sleep regulation [20] the previous findings of thalamic involvement might indicate a neural network linking WM function to sleep regulation. Such linkage is also supported by the fact that higher alertness during a WM task might help in overcoming sleepiness, a finding also positively correlated to higher fMRI-activity in the prefrontal cortices [21].

We previously investigated five individuals with fMRI and MRS during a WM task and found a correlation between the thalamic activation pattern and thalamic NAA-levels (presented at the ISMRM 2009, unpublished data). The major aim with the present study aim was to clarify if the WM deficit is due to a certain thalamic metabolic disturbance, or if the increased activation is a reflection of a deficit somewhere else in the neural networks of WM. We also aimed at investigating if there is a correlation between a possible disturbance and whether the disease is active or in remission as there are indications that memory disturbances are present even many years after remission. This could be a step in elucidating if the two demonstrated disturbances in KLS, periodic sleep patterns and WM-deficit, are correlated and if so, which one is primary and which one is secondary [6],[8]. The other parts of the brain where our previous study has shown differences in fMRI-activation, prefrontal and cingulate cortices, were not reliably assessable with the MRS-technique of our availability at the time of initiation of this study.

## Materials and Methods

### Subjects

We examined fourteen patients, six men and eight women, with median/minimum/maximum age of 20.5/14.9/37.3 years. They were diagnosed by a physician with extensive experience of KLS and fulfilled the criteria of KLS according to The International Classification of Sleep disorders, revised by the American Academy of Sleep Medicine 2005. Eight patients had an active disease, defined as at least one symptomatic period during the last one and a half year prior to the examination. The other six patients were regarded as in remission, defined as having no hypersomnia periods for 1.5 years. This interpretation was later confirmed as none of these six patients had any subsequent hypersomnia periods. Patients with active disease were examined during asymptomatic periods, i.e. they had no excessive sleepiness during examinations. No patient was on any medication interfering with CNS-activity for at least a year prior to the examinations. Demographic data are presented in Table 1.

**Table 1:** Clinical characteristics of the included patients.

No	YoB	Exam date	Gender	Symptoms	Presentation age	Triggers	Ann. freq.	Duration	Active/remission	sWmi	Handedness
1	1988	2009	f	Hs	16	A	4–5	1–2 w	r	Yes	R
2	1991	2010	m	Hs, Hph, Dp	13	none	12	1½ w	a	No	R
3	1988	2009	f	Hs, Dp	15	none	2	3–4 w	a	No	R
4	1990	2010	f	Hs, Hph, Dp, Hx, Ha	14	none	3	1–2 w	r	No	R
5	1989	2009	m	Hs, Dp	15	I	1–2	1 w	r	No	R
6	1993	2008	f	Hs, Dp, Hph	15	I, Me	2–4	1–2 w	a	No	R
7	1979	2008	m	Hs, Hx	13	none	7	1–2 w	r	No	R
8	1989	2008	f	Hs, Hph	16	A	2	2 w	r	No	R
9	1972	2008	f	Hs, Hph, Ha	15	none	6	1–2 w	a	Yes	R
10	1994	2009	m	Hs, Dp	13	none	2	4 w	a	Yes	R
11	1994	2009	m	Hs, Ps	14	I	3	1 w	a	No	R
12	1988	2009	f	Hs	15	I	2	½-1 w	a	Yes	R
13	1979	2009	f	Hs	16	none	1	2 w	a	Yes	R
14	1957	2008	m	Hs, Ps	16	none	3	2 w	a	No	R

YoB = year of birth, Ann. Freq = patient appreciated annual frequency of episodes, Duration = duration of episodes, Hs = hypersomnia, Hph = hyperphagia, Ha = hallucinations, Dp = depersonalisation and/or derealisation (as not clearly separated by some patients), Hx = hypersexuality, Ps = psychiatric symptoms, A = alcohol intake, I = infection, Me = Mental exhaustion, sWmi = subjective working memory impairment (asymptomatic periods), R = right.

As a control group, we recruited a group of 15 healthy control subjects with a median/minimum/maximum age of 22.1/18.8/40.1 years, all subjects were over 18 years of age for ethical reasons, hence a slight age mismatch.

Working memory function of all subjects was examined in a paper and pencil version of the reading-span task, by Daneman and Carpenter. The results were considered as the baseline WM function of the subjects [22]. Patients and controls were compared using a two-sample t-test.

### fMRI and Working Memory

The fMRI-paradigm was presented as previously described [9]. The paradigm consisted of a WM task with four different levels of difficulty. During fMRI-examinations, the subjects were presented with sentences using video-goggles (Resonance Technology Inc, CA, USA). Superlab Pro v 4.0 (Cedrus Corp., San Pedro, USA) was used for presenting the visual WM paradigm. Each sentence remained on the screen for five seconds. The subjects were instructed to press one button if the sentence was correct and another if it was incorrect. One to four different sentences were used in each block. After presentation of the sentences, four words were sequentially shown for 5 s each. The subjects were instructed to indicate if they recognised the word as being the last word of a previously presented sentence, or if it was a new word, not shown before.

All MR measurements were performed using an Achieva 1.5 T MR scanner (Philips, Best, The Netherlands). Functional images were acquired using BOLD-EPI sequence and the standard head coil. The MR-imaging parameters were: echo time (TE) = 40 ms, repetition time (TR) = 2.7 s, flip angle 90°. Thirty-two slices without slice gaps were obtained in interleaved mode. Voxel size was 3×3×3 mm^3^ and the number of dynamics (image volumes) was 302.

The BOLD images were preprocessed and analyzed using the SPM5 software (Wellcome Department of Imaging Neuroscience, University College, London, UK). The images were realigned to correct for movement during scanning, normalized and re-sliced to a standard MNI (Montreal Neurological Institute) template. The normalized images were smoothed with 8 mm Gaussian kernel to ameliorate differences in intersubject localization. Furthermore, the images were analyzed employing a General Linear Model (GLM) and a parametric contrast tapping the different difficulty levels at word recognition after 1–4 presented sentences.

Differences in thalamic activation between KLS patients and healthy subjects were estimated by region of interest (ROI) analysis. The ROI was identified as the significant activation cluster for KLS patients in the left thalamus, which was reported in our previous study [9]. The ROI center of mass was −6; −9; 6 and the volume was 944 mm^3^. Difference images were obtained by exclusively masking the KLS activation map with the map of healthy controls (mask p-value = 0.05). In the whole brain analysis, an uncorrected threshold of p = 0.001 was used to obtain the activation maps. Results are reported as significant if cluster p<0.05, family wise error (FWE) corrected. In addition, differences in thalamic activation between KLS patients and controls were estimated by region of interest (ROI) analysis. The ROI was identified as the significant activation cluster for KLS patients in the left thalamus, which was reported in our previous study [8]. The ROI center of mass was −6; −9; 6 (MNI coordinates) and the volume was 944 mm^3^. The significance threshold for the ROI analysis was chosen to be p = 0.05, corrected for family wise error (FWE). Finally, the individual activation levels in the thalamic ROI were calculated as the contrast estimate from non-smoothed images using the MarsBaR toolbox [23]. The individual activation levels during the most difficult condition of the working memory task, which is word recognition after four presented sentences, were calculated.

### Magnetic Resonance Spectroscopy

#### Data Acquisition

**Figure 1:**
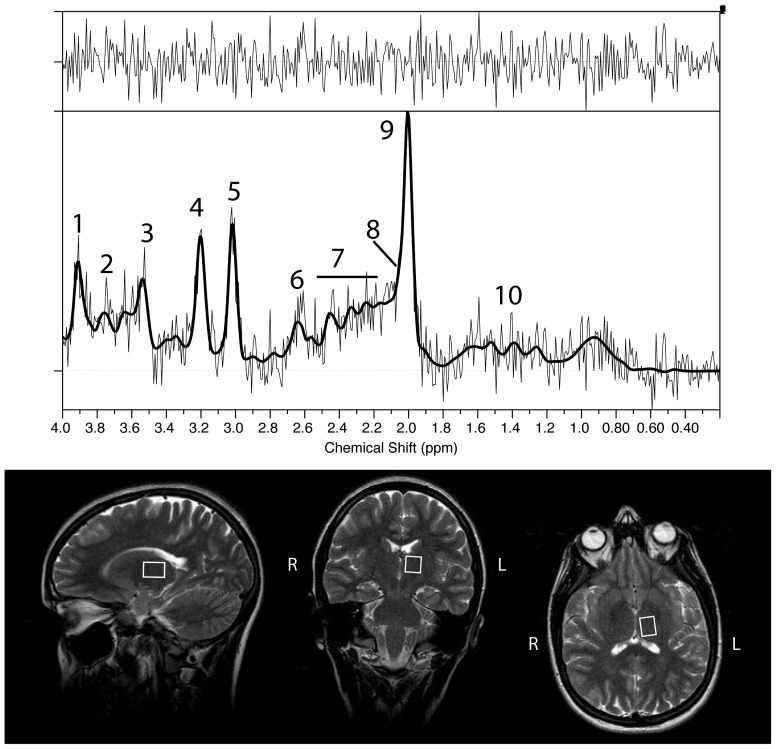
Voxel in MRS-measurement, placed in the left thalamus and a typical MRS-spectrum. Assignments of spectral resonances: 1, total Creatine (−CH_2_−); 2, Glutamine and Glutamate (CH-α); 3, myo-inositol; 4, total Choline ((−CH2)3); 5, total Creatine (−CH_3_); 6, N-acetylaspartate (CH_2_); 7, Glutamine and Glutamate (CH-γ/β); 8, N-acetylaspartate-glutamate (CH_3_); 9, N-acetylaspartate (−CH_3_); 10, Lactate.

The proton MRS data were acquired using the transmit-receive head coil and point-resolved spectroscopy (PRESS) with TE of 25 ms and TR 3 s, 128 water suppressed spectra were averaged, acquisition time 6:54 min∶sec, and two MRS volume of interest (VOI) were placed bi-laterally in left and right thalamus (TH) (Fig. 1), the volume o the VOI were adjusted to fit the individual anatomy of each subject with a volume of ca. 3.00 mL. To detect a possible systemic variation of the metabolites we also placed a MRS VOI in the frontal white matter bi-laterally. In the KLS group the min/median/max thalamus volume was 2.02/2.27/3.14 mL. In the control group the min/median/max was volume 2.02/2.27/2.86 mL. T2w coronal images were acquired prior to and after each MRS acquisition in order to determine patient movements. Whole brain coverage quantitative MRI (qMRI) data were acquired using QRAPMASTER sequence with a resolution of 3×1×1 mm^3^ [24], four echoes and four dynamics using TE = 20.81 ms, TR = 3.7 s. acquisition time 7:05. Min∶sec.

#### Processing of MR data

The MRS-data were analyzed using LCModel ver. 6.2-1T [25]. The unsuppressed water signal was used as an internal concentration reference and it was quantified using the water scaling function in LCModel, with the ‘attenuation-of-NMR-visible-water’ (ATTH2O) adjusted to 1.00 (instead of default 0.7). Thus the resulting concentrations were obtained and presented with respect to the aqueous concentrations and completely compensated for by the differences in coil load and temperature, and difference in RF amplification etc. in different subjects. However, the concentrations determined in this manner also depend on the magnitude of the relaxation of the unsuppressed water signal. In order to correct for the relaxation effect, the qMRI-volume were used for this purpose. The qMRI data were processed using SyMRI Brain Studio ver 2 software (SyntheticMR, Linköping, Sweden). Quantitative maps of R_1_ ( = 1/T_1_), R_2_ ( = 1/T_2_) and PD covering the brain, were exported as DICOM stacks for further analysis. A multiplicative scaling factor ‘*f_scale_*’ was estimated for each MRS (Eq. 1).
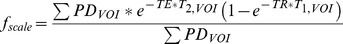
(1)Where PD_2,VOI_, R_1,VOI_ and R_2,VOI_ were the qMRI values within the MRS VOI. Using this scaling factor the estimated concentrations were converted to wet-weight concentrations using the unit mM of aqueous fraction (mM aq.) [26].

#### Group Statistic Analysis: MRS

JMP 8 (SAS Institute Inc, USA) was used to calculate the group statistical analysis. Mean concentrations and standard errors of mean were estimated using a full mixed linear model with the three fixed factors ‘group’, ‘lateral’, ‘anatomical structure’ and all crossing effects. Difference in mean concentrations were then tested using a Student's t-test and p-values<0.05 were considered significant.

#### Correlation Analysis: MRS and fMRI

The values of the effect sizes in the left thalamus derived from fMRI were correlated to the estimated metabolite concentration of the left and right thalamus using GraphPad Prism 5.0a (GraphPad Software, San Diego California USA) Pearson correlation, p-values<0.05 were considered significant.

### Ethics Statement

The study design was approved by the Regional Ethical Approval Board according to the Swedish Ethical Review Act (2003) in accordance with the Helsinki Declaration of 1975.

## Results

**Figure 2:**
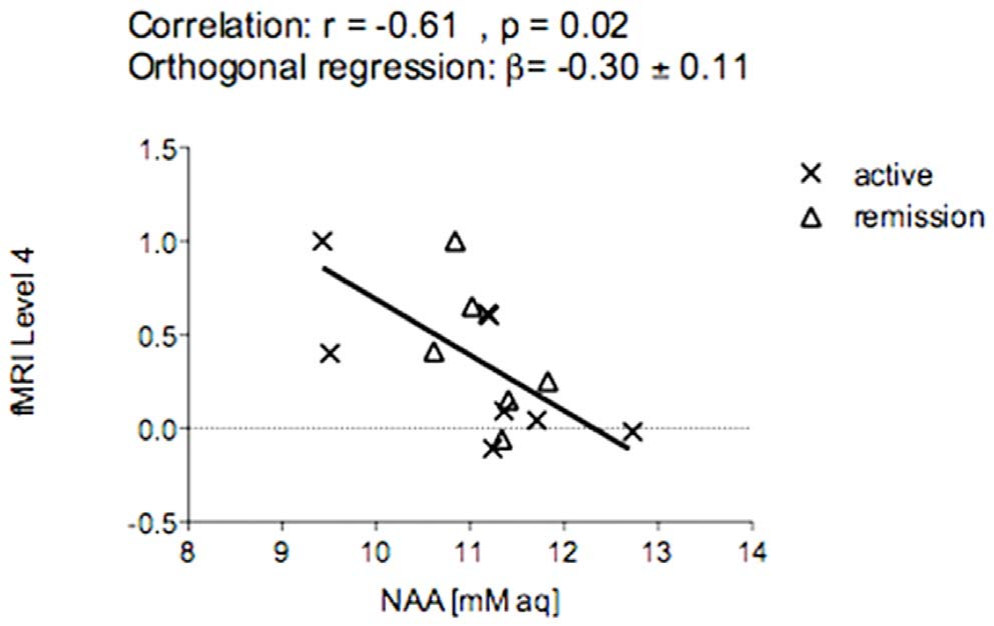
Regression analysis of fMRI-activiation and absolute NAA-concentraion in the left thalamus of KLS-patients. [mM aq] = mM of aqueous fraction.

The main finding of this study was a negative correlation between thalamic activation patterns in fMRI (at the most difficult level in the WM task) and the left thalamic absolute NAA concentration in KLS patients (r = −0.61, p = 0.022) as illustrated in Figure 2. In contrast, no significant correlation between thalamic activation patterns in fMRI and the left thalamic absolute NAA concentration in healthy controls was observed (r = 0.45, p = 0.14). fMRI data from 3 healthy controls were excluded from the correlation analysis due to head movement during fMRI acquisition.

In the pen and paper working memory reading span task, patients recalled less words than the healthy controls; mean 16 (SD 3.4) vs 22 (SD = 5.7), p = 0.01. In the fMRI-task, healthy controls scored slightly more correct words than patients (p = 0.01) at level 4 but not at level 1–3 (Table S1).

The fMRI whole brain level analysis confirmed earlier published results of larger activation volume in the left thalamus and the left lateral prefrontal cortex in KLS patients compared to healthy controls during the WM task. However, in the current study, we found the main cortical activation difference in the opercular part of the left inferior frontal gyrus (−42 4 28, Z = 4.72, cluster size = 442) rather than in the triangular part, as reported earlier. KLS patients also had significantly larger activation volume in the bilateral parietal cortex compared to controls: 633 voxels in the left hemisphere with peak at −26 −74 52 (Z = 4.82) and 631 voxels in the right hemisphere with peak at 28 −66 50 (Z = 4.97). These bilateral parietal clusters encompassed both superior–medial and inferior–lateral aspects of the parietal cortex. Further, in this study we observed that KLS patients had significantly more activation in the bilateral caudate (−16 2 12, Z = 4.26, cluster size = 160; 14 4 12, Z = 4.05, cluster size = 132). The cluster in the left caudate extended into the left thalamus (see below). In addition, the KLS patients had more activation in the bilateral occipital cortex compared to controls. The controls did not have larger activation volume compared to KLS in any cortical areas.

**Figure 3:**
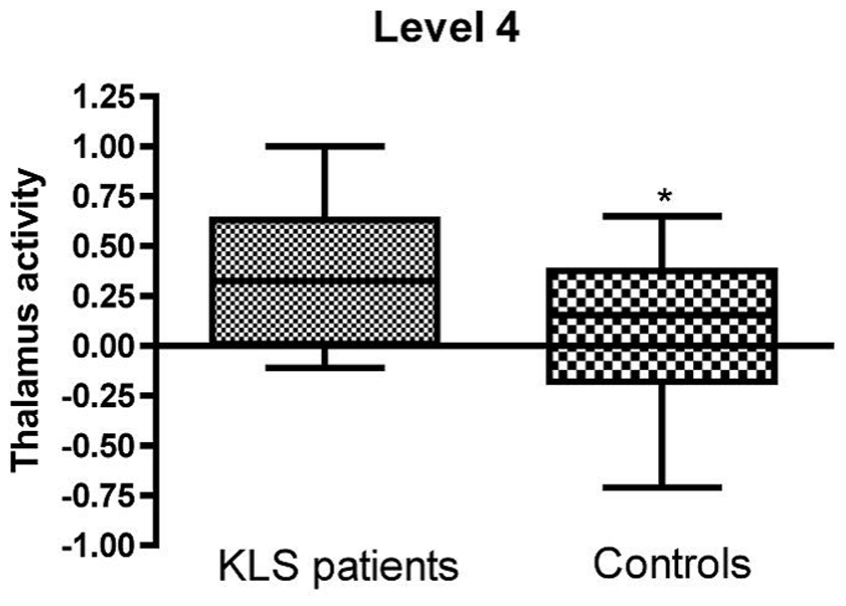
Differences in fMRI-activation of the left thalamus at the most difficult level (Level 4) of the WM task in KLS-patients and healthy control subjects. p = 0.04 (t-test).

**Figure 4:**
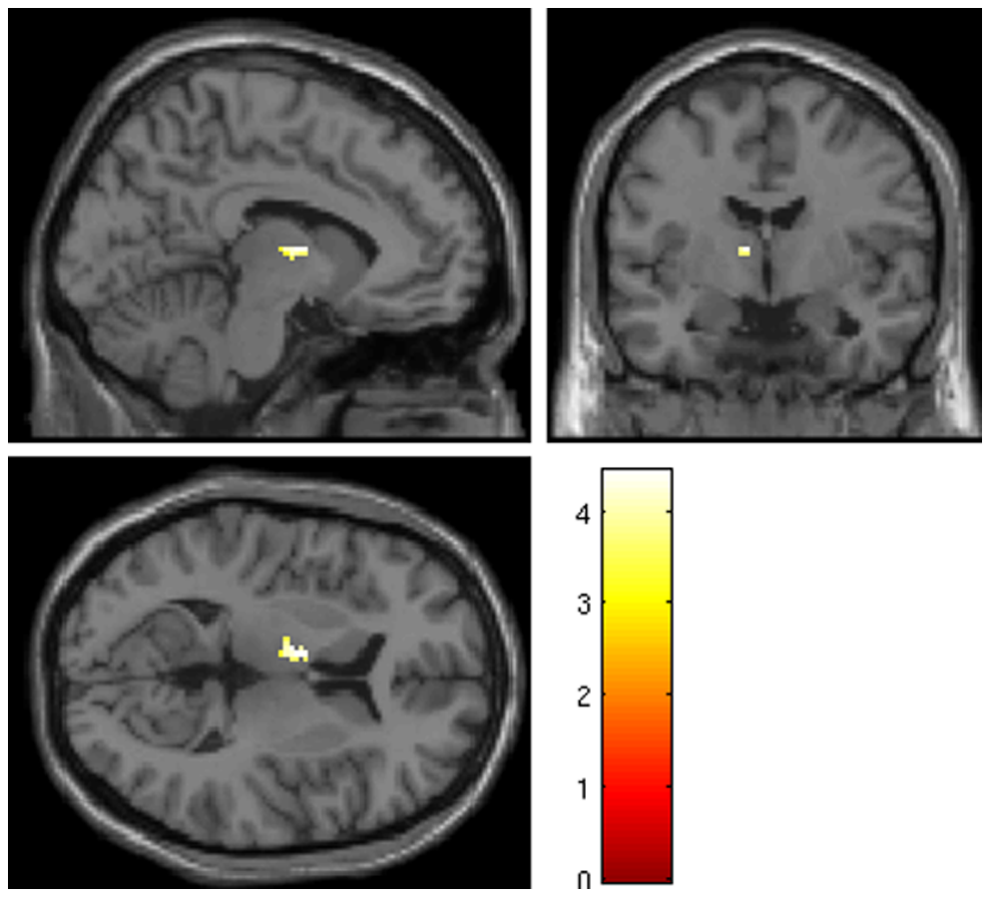
Region of interest analysis in the left thalamus showing significant difference in KLS compared to control subjects.

Importanly, the fMRI results confirmed the earlier published differences in thalamic activation between KLS-patients and healthy control subjects (Figures 3 and 4). On a group level, KLS-patients had significantly (cluster p = 0.003, peak p = 0.004, FWE corrected) increased activation in the left thalamus (peak at −8 −4 8, Z = 3.75, cluster size = 31 voxels) during the WM task. As shown in Figure 2, the individual variation of the BOLD signal in the thalamus of KLS-patients is correlated to NAA levels. In contrast, the interindividual variation in the control group was neither correlated to NAA levels, or to the WM task result. Thus, no fMRI-activity significantly different from background activity was detected in healthy controls, as was the case in the left thalamus of the KLS-patients [9].

To test the validity of the correlation in the entire KLS group vs. the previously tested group of five individuals (ISMRM 2009), as a control experiment, we repeated the analysis excluding these patients. Exclusion of these patients still resulted in a negative correlation (p = 0.022).

A similar regression analysis for other metabolites showed no significant correlation in any of the groups. In contrast, the correlation was not observed if the analysis was performed using the NAA/Cr-ratio as a relative measure rather than absolute NAA-concentration.

The differences in fMRI activation patterns were not reflected by any significant differences in metabolite concentrations on a group level in either patients or controls as illustrated in Table 2 and Table 3. Neither were there any differences between left and right thalami within the groups resulting with similar metabolite concentrations for all four areas measured (right and left thalami in patients and controls respectively). For NAA the mean concentration in the KLS group was 11.33 mM aq. (SE 0.24) and 11.12 mM aq. (SE 0.24) for left and right thalami, respectively, and 11.10 mM aq. (SE 0.25) and 11.15 mM aq. (SE 0.25) for the healthy controls. In frontal white matter, no differences where observed between the groups concerning concentration of any of the metabolites measured.

**Table 2:** Group mean metabolite concentrations on MRS.

	Controls				KLS			
	TH-L		TH-R		TH-L		TH-R	
	Mean	SE	Mean	SE	Mean	SE	Mean	SE
tCr	7.60	0.19	7.84	0.19	7.69	0.19	7.76	0.19
NAA	11.33	0.24	11.12	0.24	11.10	0.25	11.15	0.25
tNA	12.54	0.26	12.38	0.26	12.52	0.27	12.50	0.27
mIns	5.12	0.32	5.30	0.32	4.83	0.33	4.73	0.33
tCho	2.44	0.08	2.48	0.08	2.29	0.09	2.25	0.09
tGlx	15.99	0.53	15.85	0.53	14.37	0.55	16.02	0.55
NAA/tCr	1.50	0.05	1.43	0.05	1.45	0.05	1.46	0.05
tNA/tCr	1.66	0.06	1.60	0.06	1.64	0.06	1.63	0.06
mIns/tCr	0.32	0.01	0.32	0.01	0.30	0.01	0.29	0.01
tCho/tCr	0.67	0.05	0.68	0.05	0.64	0.05	0.60	0.05
tGlx/tCr	2.12	0.11	2.06	0.11	1.89	0.11	2.08	0.11

Group mean concentrations and standard error (SE) for metabolites determined by magnetic resonance spectroscopy (MRS). Absolute concentrations are given in mM of aqueous fraction (mM aq.). tCr = total creatine, NAA = n-acetylaspartate, tNA = NAA+ n-acetylaspartateglutamate, mIns = myo-inositol, tCho = total choline, tGlx = glutamate+glutamine. TH-L = left thalamus. TH-R = right thalamus.

**Table 3:** Differences in group mean metabolite concentrations on MRS.

	KLS TH-L - Controls TH-L			KLS TH-R vs. Controls TH-R			KLS TH-L vs. KLS TH-R			Controls TH-L vs. KLS TH-R		
	Diff	SE	p	Diff	SE	p	Diff	SE	p	Diff	SE	p
tCr	0.088	0.270	1.000	−0.081	0.270	1.000	−0.070	0.274	1.000	−0.240	0.264	0.985
NAA	−0.227	0.343	0.998	0.031	0.343	1.000	−0.049	0.314	1.000	−0.209	0.303	0.997
tNA	0.013	0.378	1.000	0.122	0.378	1.000	0.026	0.343	1.000	0.161	0.332	1.000
mIns	−0.291	0.463	0.998	−0.563	0.463	0.925	0.094	0.398	1.000	−0.178	0.385	1.000
tCho	−0.147	0.118	0.917	−0.234	0.118	0.504	0.042	0.090	1.000	−0.045	0.087	1.000
tGlx	−1.616	0.765	0.413	0.167	0.765	1.000	−1.644	0.771	0.404	0.140	0.745	1.000
NAA/tCr	−0.046	0.075	0.999	0.023	0.075	1.000	−0.001	0.072	1.000	0.069	0.070	0.976
tNA/tCr	−0.024	0.090	1.000	0.032	0.090	1.000	0.004	0.088	1.000	0.059	0.085	0.997
mIns/tCr	−0.037	0.067	0.999	−0.075	0.067	0.952	0.035	0.056	0.999	0.003	0.054	1.000
tCho/tCr	−0.020	0.020	0.974	0.030	0.020	0.811	0.012	0.017	0.997	0.002	0.016	1.000
tGlx/tCr	−0.229	0.151	0.798	0.024	0.151	1.000	−0.188	0.151	0.916	0.065	0.146	1.000

Differences in group mean metabolite concentrations as determined by magnetic resonance spectroscopy (MRS). SE = standard error. tCr = total creatine, NAA = n-acetylaspartate, tNA = NAA+ n-acetylaspartateglutamate, mIns = myo-inositol, tCho = total choline, tGlx = glutamate+glutamine. TH-L = left thalamus. TH-R = right thalamus.

The distribution of patients in the regression analysis did not show any clustering related to active or remitted disease as illustrated in Figure 2.

## Discussion

The finding of a negative correlation between thalamic fMRI-activity and NAA-concentration in the KLS-group and the absence of such a significant variation of fMRI-activity in healthy subjects suggest that there is no simple connection between the concentration of thalamic neurons and the level of thalamic activity during a WM task. The finding can be interpreted as the effect of an effective neurological difference between the KLS patients and healthy controls, for example differences in the dynamics of the networks involved in performing the WM task.

The NAA/Cr-ratios were comparable to the ratios described by Poryazova *et al* in asymptomatic periods, i.e. 1.3–1.5, albeit they used somewhat different acquisition conditions [18].

A direct comparison between the groups has to take into account the fact that the healthy individuals did not show significantly increased thalamic activity on fMRI. As the patient group showed increased activation upon higher WM task difficulty levels, we hypothesize, based on the findings, that the previously described WM deficit [9] influences the effort needed to complete the task at lower levels. This could imply that with more complex WM tasks, the healthy controls would have a similar activation pattern, possibly resulting in comparable negative correlations to NAA-concentrations. This is supported by the fact that more complex WM tasks have resulted in more complex PET and fMRI activation patterns also in healthy subjects, including increased activity of the thalamus and prefrontal cortices [27]–[31]. Such an increase can be attributed to a greater need for involvement of the visual attention networks, *i.e.*, the subject has to concentrate harder while performing a more difficult WM task. Neuro-anatomically, “frontal–subcortical circuits” for WM is a well-established concept. These circuits describe projections from the prefrontal cortex to the striatum, globus pallidus, substantia nigra and the thalamus. The thalamus have projections back to the prefrontal cortices forming several loops. These loops have been shown to be involved in both WM and visual attention [32]. The networks of verbal working memory and visual attention both activate the thalamus, but if the two functions are tested more selectively and with increased WM and visual attention loads, they differ in activation patterns with WM activating more of the prefrontal cortices. This has been interpreted as a need to deploy more executive resources with increasing WM-load and this finding indicates that activation patterns of the networks for WM and visual attention depend on task difficulty [33]. The KLS-patients have not been tested exclusively for visual attention, but in our previous and current studies they show more activation in the left lateral prefrontal cortex, previously shown to be more exclusively attributed to WM [27]. As stated above, the activation patterns in KLS-patients is probably due to an increased effort at lower load, an interpretation that can support a deficit in either of the two principal components of working memory, *i.e.* working memory and visual attention networks, this since both the thalamus and the prefrontal cortex are increasingly activated in the patient group.

Another important issue to address is the role of NAA in relation to neurons and neuronal function. The somewhat vague terms of “neuronal health, viability and number” was thoroughly reviewed by Moffet *et al.* in 2007 [13]. NAA is synthesized from aspartate and acetyl-coenzyme A, a metabolism that to this date has been detected in neurons exclusively. In contrast, a theory of NAA having a role in axon-glial signaling has also been proposed; such a role is supported by the fact that catabolic enzymes are located in oligendendrocytes and astrocytes. The theory emphasizes that NAA, which is partly metabolized in glial cells, re-cycle to the neurons, thereby accentuating the neuronal specificity and role of NAA. Even if this inter-cellular metabolism does not show a variability throughout the CNS, there is still a variation of NAA concentration. This is important as it shows that NAA may not be proportionally related to neuronal numbers. As the NAA-absolute quantification method used in this publication estimates NAA based on the water fraction (mM aq.), we prefer the term “neuronal concentration” to the term “neuronal number”. This is supported by the fact that other, non-neuronal specific, metabolites do not covariate with NAA, indicating that the variation is not a result of increased tissue water content, or partial volume effects. The non-specific terms “neuronal health and viability” might nevertheless be partly justified for NAA. A neuronal specific TCA (tricyclic acid) cycle has been proposed, directly linking NAA to the mitochondrial energy metabolism. This, in turn, links NAA to glutamate, which is also dependent on asparate aminotransferase [13],[34],[35]. As glutamate is proposed to have a significant role in the changing BOLD-signal, stimulating neurons and glial cells to release vascular dilating factors [36], [37], aspartate aminotransferase might be linking NAA indirectly to BOLD-signal. Recently, the role of GABA, acting trough inhibitory interneurons or directly on the vasculature, has been a subject of increasing interest, though it has not been shown to be linked with the NAA-concentrations [38].

With respect to the thalamus, several previous studies have implicated that it is increasingly activated with increased WM demand [29]–[31]. This has also been shown to be connected to sleep deprivation/state of sleepiness [39]. Other studies have shown that in other disorders with WM deficits, such as normal pressure hydrocephalus, the thalamic absolute NAA-concentrations decrease [15]. To the best of our knowledge, no previous group studies have coupled such findings on fMRI and MRS on an individual level.

The significant correlation between absolute NAA-concentration and fMRI-activation, in combination with the lack of such correlation to the NAA/Cr-ratio, implies that the variability of the ratio described by Poryazova *et al.* reflected a temporal variability in Cr-concentration, rather than a variation of NAA concentration [18]. A variation of NAA-concentration – which primarily represents neuron concentration – is indeed more difficult to accept, taking the above discussion on NAA as a fraction of water (mM aq.) in account and comparing it to the lack of variation of other metabolites.

Some of the limitations of this study were partly due to technical limitations, while others were apparent only after the analysis of the results. Limitations in the MRS-technology at the time of the study design excluded a parallel analysis of the cortical areas of interest in the fMRI-results. The absence of a significant difference in thalamic levels of NAA between patients and controls, despite the finding of a correlation, would argue against a primary thalamic dysfunction. On the other hand, the use of a voxel of interest in the MRS study including the entire thalamus and not only the anterior-medial parts (where an increased activation was evident using fMRI) may have affected the results. This could be a confounding factor as the individual variation of NAA-concentrations in both patients and controls could reflect an individual variation in the actual distribution of the thalamic neurons. Chee and Choo emphasized that WM-function probably engages specific thalamic areas [40]. Thus, the KLS-patients that had high neural activity and low NAA-concentrations, might have a NAA-distribution that does not correlate to the regions involved in pure WM neural networks. Another aspect for interpreting these results is the relatively small number of metabolites detectable on MRS, only metabolites with concentration exceeding c. 0.5 mM will be detected using standard acquisition procedures on clinical MR-scanners. Neural function depends both on neurotransmittors and their receptors, but also on the neural connectivity, the latter is not examined using these methods. It would be of great interest to find other correlates to the findings, such as clinical data like disease duration. Additionally, it would be interesting to test attention and WM networks more selectively and extensively and to relate them to MRS and fMRI findings.

In summary, the main conclusion is that there is a significant negative correlation between thalamic fMRI-activity and thalamic NAA-concentration in patients with Kleine Levin Syndrome (KLS), a group of patients in which we previously have been able to demonstrate a disturbed WM performance. In contrast, this correlation was not observed in healthy control subjects. This implies that the KLS-patients have a pathological disturbance somewhere in the neural network responsible for WM-performance, which results in an increased activity in the thalamus.

To further elucidate exactly where in these networks this disturbance is present – further investigations will be required. Possible foci for such investigations are prefrontal cortices, cingulate cortex, and the basal ganglia. As implicated above, it would also be of great interest to examine other metabolites, such as glutamate and GABA, in relation to working memory performance, with more selective and sensitive MRS procedures. Certainly, it would also be most interesting to investigate if the fMRI activation patterns of KLS-patients are reproducable in healthy control subjects in more difficult working memory tasks.

## Supporting Information

Table S1 **Working memory performance during fMRI.**
(DOCX)
