# Supplementary material for: Low Thalamic NAA-Concentration Corresponds to Strong Neural Activation in Working Memory in Kleine-Levin Syndrome
Source: PLoS One. 2013 Feb 25;8(2):e56279. doi: 10.1371/journal.pone.0056279 (PMC3581507; doi:10.1371/journal.pone.0056279)
Supplement: Table S1 — Working memory performance during fMRI. (DOCX) [file pone.0056279.s001.docx]

**SUPPLEMENT**

**Table S1: Working memory performance during fMRI**

The table shows the mean hit rates calculated as the percentage of correct responses during each level of difficulty of the working memory task. The standard deviations are given in parenthesis. The difference between groups was significant at Level 4, p=0.01. Differences between the other levels were not significant.

|  | **Level 1** | **Level 2** | **Level 3** | **Level 4** |
| --- | --- | --- | --- | --- |
| **KLS** | 0.97 (0.04) | 0.95 (0.08) | 0.95 (0.10) | 0.91 (0.10)* |
| **Controls** | 0.99 (0.02) | 0.96 (0.05) | 0.99 (0.02) | 0.99 (0.01) |
